# 线粒体在铁死亡中的作用及其与肿瘤的关系

**DOI:** 10.3779/j.issn.1009-3419.2024.102.34

**Published:** 2024-10-20

**Authors:** Zhanrui ZHANG, Hongyu LIU, Jun CHEN

**Affiliations:** ^1^832003 石河子，石河子大学医学院第一附属医院胸外科; ^1^Department of Thoracic Surgery, First Affiliated Hospital, School of Medicine, Shihezi University, Shihezi 832003, China; ^2^300052 天津，天津医科大学总医院，天津市肺癌研究所，天津市肺癌转移与肿瘤微环境重点实验室; ^2^Tianjin Key Laboratory of Lung Cancer Metastasis and Tumor Microenvironment, Tianjin Lung Cancer Institute, Tianjin Medical University General Hospital, Tianjin 300052, China; ^3^300052 天津，天津医科大学总医院，肺部肿瘤外科; ^3^Department of Lung Cancer Surgery, Tianjin Medical University General Hospital, Tianjin 300052, China

**Keywords:** 铁死亡, 线粒体, GPX4, 脂质过氧化, Ferroptosis, Mitochondria, GPX4, Lipid peroxidation

## Abstract

铁死亡是近年来新发现的一种区别于凋亡的细胞死亡形式，以细胞内铁的累积和脂质过氧化水平增加为主要特征。肿瘤细胞对铁死亡的抵抗会促进肿瘤的发生发展；多种化合物能通过触发铁死亡来影响肿瘤进展。铁死亡存在复杂的调控机制，而线粒体作为铁的储存中心与代谢中心，对铁死亡起重要的调控作用。本文就铁死亡及其三个阶段、线粒体对铁死亡的调控机制以及铁死亡在肿瘤发生发展与治疗中的作用做一综述。

## Abstract (en)

Ferroptosis is a recently discovered form of cell death that is distinct from apoptosis, characterized primarily by the accumulation of intracellular iron and increased levels of lipid peroxidation. Resistance of tumor cells to ferroptosis can promote tumorigenesis and tumor progression. Various compounds can influence tumor development by triggering ferroptosis. Ferroptosis involves complex regulatory mechanisms, with mitochondria serving as both an iron storage and metabolic center, playing a crucial regulatory role in ferroptosis. This review discusses ferroptosis and its three stages and the role of ferroptosis in tumorigenesis, progression, and treatment, as well as the regulatory mechanisms of mitochondria in ferroptosis.

肿瘤是全球仅次于心血管疾病的导致人类死亡的第二大疾病。据世界卫生组织/国际癌症研究机构（World Health Organization/International Agency for Research on Cancer, WHO/IARC）估计，2022年全球有2000万例新增肿瘤病例和970万例死亡病例^[1]^。预计2050年将有超过3500万例新发癌症病例，比2022年估计的2000万例增加77%。根据WHO与IARC发布的数据^[2]^，预计2022年中国约482万人新诊断为癌症，其中321万人死于癌症。此外，根据美国癌症协会2024年发布的数据^[3]^，预计2024年美国将新增癌症病例200万例，癌症死亡病例612万例。其中肺癌发病率在中国位居第一，在美国位居第二，死亡率在中国与美国均为第一。

细胞存在多种调节性死亡形式，如凋亡、细胞程序性死亡、焦亡等。铁死亡是近年备受关注的细胞调节性死亡形式，主要受磷脂过氧化损伤和细胞内铁代谢调控。铁死亡参与肿瘤、神经退行性疾病和缺血/再灌注损伤等多种疾病的进程^[4]^。研究^[5]^指出，肿瘤细胞对铁死亡的抵抗，会促进肿瘤的发生发展；此外，多种化合物和抗癌药物能够通过触发铁死亡来影响癌症进展^[6,7]^。因此，研究铁死亡的调控机制以及如何利用药物靶向铁死亡途径从而有针对性地治疗不同类型的肿瘤已经逐渐成为肿瘤研究的热点问题之一^[8]^。

铁死亡有着复杂的调控机制，包括最经典的谷胱甘肽过氧化物酶4（glutathione peroxidase 4, GPX4）调节的铁死亡途径，以及GPX4以外的其他调控途径，越来越多的基因被发现参与铁死亡的调控^[9]^。线粒体与铁死亡有着复杂的联系，本文就铁死亡及其三个阶段、线粒体对铁死亡的调控机制以及铁死亡在肿瘤发生发展与治疗中的作用做一综述。

## 1 线粒体与肿瘤

线粒体是细胞内重要的能量工厂，主要由外膜、内膜和基质构成。内膜的折叠形成嵴，增加其表面积，从而提高了ATP的合成效率。线粒体的功能包括进行氧化磷酸化以提供能量、脂质代谢、储存钙离子、参与细胞凋亡以及产生活性氧（reactive oxygen species, ROS）以调控信号传递^[10]^。正常的线粒体功能对细胞代谢至关重要，其障碍与多种神经退行性疾病及肿瘤的发生密切相关^[11]^。

代谢重编程是肿瘤细胞的重要特征，主要表现为糖酵解增强、谷氨酰胺代谢活跃及脂质代谢异常^[12]^，这一现象被称为瓦博格效应。尽管瓦博格提出线粒体呼吸缺陷是有氧糖酵解的原因，但并非所有肿瘤均表现此特征。许多癌症中的致癌基因突变（如K-ras、c-Myc和p53）才是有氧糖酵解的主要驱动因素。研究^[13]^发现，p53蛋白的突变会导致葡萄糖-6-磷酸脱氢酶的活性失调，进而过度激活磷酸戊糖途径，这为肿瘤细胞的增殖和DNA复制提供原料。

线粒体在调控细胞死亡方面发挥重要作用，尤其是通过调节凋亡过程。当细胞受到凋亡刺激时，线粒体膜通透性发生改变，导致细胞色素C和凋亡诱导因子等蛋白释放到细胞质中，激活半胱氨酸天冬氨酸蛋白酶（cysteinyl aspartate specific proteinase, Caspase），引发细胞凋亡。Bcl-2家族蛋白在此过程中起关键作用^[14]^，促凋亡蛋白（如Bax和Bak）促进线粒体膜通透性增加，而抗凋亡蛋白（如Bcl-2和Bcl-xL）则维持膜的稳定性。此外，线粒体还通过调节钙离子稳态和自噬作用影响细胞存亡。当钙离子浓度异常时，线粒体功能受损，可能导致细胞凋亡。自噬则通过选择性清除受损线粒体，维持细胞稳态。综上所述，线粒体不仅是能量代谢的中心，也是肿瘤发生和细胞死亡调控的重要参与者。

## 2 铁死亡及铁死亡的三个阶段

### 2.1 铁死亡发生的第一阶段：ROS的生成

ROS是一类含氧的化合物，可以在细胞内参与电子转移反应。它的种类包括O^2-^、H_2_O_2_、^-^OH、^1^O_2_、NOX、RNS等^[15]^。O^2-^的产生与线粒体代谢电子泄露有关，线粒体的多个位置可能发生电子泄漏^[16]^，泄露的电子与O_2_发生氧化反应，形成O^2-^。线粒体中至少有12个场所可产生O^2-^，包括电子传递链复合物以及某些线粒体脱氢酶^[17]^。线粒体ROS主要来源是电子传递链复合物I和III^[18]^。O^2-^会在超氧化物歧化酶（super oxide dismutase, SOD）的作用下迅速转化为H_2_O_2_。

H_2_O_2_的反应性并不强烈，寿命较长。它可以通过膜扩散，因此可以在远离其产生部位的地方产生影响。在所有的ROS中，H_2_O_2_主要参与蛋白质氧化。虽然低水平（1-10 nmol/L）H_2_O_2_在氧化还原信号[蛋白酪氨酸磷酸酶（protein tyrosine phosphatase, PTP）、胰岛素]的传导中起重要作用，但较高水平（100 nmol/L）的H_2_O_2_会导致“氧化应激”^[19]^。

由于O^2-^与H_2_O_2_的反应并不强烈，大多数ROS诱导的细胞损伤是由于它们转化为其他物质导致。^-^OH在所有ROS中反应性最强。H_2_O_2_在Fe^2+^的作用下会发生芬顿反应，生成^-^OH。现在已经具备检测细胞的ROS水平的能力，但将不同的ROS区分进行检测依然是个挑战。

### 2.2 铁死亡发生的第二阶段：Fe^2+^的累积

铁离子通常以三价铁形式与转铁蛋白（transferrin, TF）结合，通过TF通道进入细胞，被金属还原酶家族——前列腺六跨膜上皮抗原3（six-transmembrane epithelial antigen of the prostate 3, STEAP3）还原成二价铁后，参与后续多种生理和生化过程。进入细胞的铁被还原成亚铁离子，优先形成各种铁结合复合物，参与各项生理和生化反应。铁结合复合物含量接近饱和时，多余的二价铁会在细胞中积累，形成不稳定铁池。铁池中游离的二价铁参与芬顿反应，生成以羟基自由基为代表的ROS，积累的ROS引起膜脂过氧化，从而导致细胞功能丧失和细胞死亡。这是细胞铁死亡的经典调控途径之一^[20]^。不难看出，铁代谢对铁死亡的调控主要在于对不稳定铁池的调控，增加铁的输入、降解铁蛋白都可以使铁池不稳定铁含量增加，增加铁死亡的风险；而增加铁的输出、合成铁蛋白会降低铁含量，可以预防铁死亡。

控制铁元素的输入是调控细胞内铁池容量的主要途径之一。铁离子通过TF/TF受体-1转运系统（TF/TF receptor-1 transport system, TF/TFR-1）或溶质载体家族39成员14（solute carrier family 39 member 14, SLC39A14）进入细胞，相关蛋白表达水平的提升或对于TFRC[TF受体1（TF receptor 1, TFR1）编码基因]的过度激活都会导致细胞内铁离子过载，在不同程度上诱导铁死亡发生。而热休克蛋白β1（heat shock protein beta-1, HSPB1）的表达可以抑制TFR1的表达水平^[21]^，减少铁的摄入，控制铁池容量。

将铁池中不稳定状态的铁合成各种含铁蛋白，是细胞内铁离子的重要应用途径。细胞中游离的铁元素在铁调节蛋白的作用下被各种生理和生化功能所利用，包括合成铁硫蛋白等多种铁结合蛋白，或形成铁蛋白成为贮存铁。研究^[22]^发现，三四脯氨酸（trans-4-hydroxyproline, Trans-4-OHP）在细胞缺铁条件下表达，能通过降解mRNA转录物，减少多种与铁结合的蛋白尤其是铁硫蛋白的合成，维持细胞铁池的容量。铁反应元件结合蛋白（iron responsive element binding protein, IRE-BP）的表达会增加铁蛋白重链和轻链的合成，诱导铁离子形成稳定状态。

通过降解细胞内的含铁蛋白来回收铁离子，也是增加细胞内不稳定铁池容量的途径之一，如核受体共激活因子4（nuclear receptor coactivator 4, NCOA4）能通过对铁蛋白进行选择性自噬来释放铁蛋白中的铁离子；而核因子红细胞2相关因子2（nuclear factor erythroid 2-related factor 2, NRF2）调控的血红素加氧酶1（heme oxygenase 1, HO-1）能催化血红素降解产生亚铁离子^[23]^。

将细胞中的铁转运至细胞外，也是控制和维持细胞铁稳态的途径之一。如铁转出蛋白和铁蛋白转出蛋白可分别将铁离子和铁蛋白通过各种途径输送至细胞外。其表达的降低已被证实会促进铁死亡的发生^[24]^。直接使用铁螯合剂来清除不稳定状态的铁，也是铁死亡研究中的常用方式之一。

### 2.3 铁死亡发生的第三阶段：脂质过氧化

多不饱和脂肪酸（polyunsaturated fatty acids, PUFAs）含有活性氢原子，其对脂质过氧化高度敏感，这破坏了细胞膜脂质双层的完整性从而引起了细胞死亡。脂质过氧化由^-^OH引发，反应生成的H_2_O_2_在Fe^2+^引发的芬顿反应下继续生成^-^OH，导致链式反应。在Fe^2+^的依赖下过度的脂质过氧化引发的细胞死亡称为铁死亡^[25]^。

磷脂氢过氧化物（phospholipid hydroperoxides, PLOOHs）作为铁死亡执行者的作用现已确定。PUFA链中的二烯丙基（^-^CH=CH-CH_2_-CH=CH^-^）是反应发生的结构基础，由于周围双键的作用，二烯丙基在自由基的攻击下会异构化为更稳定的共轭二烯转态并生成新的自由基，最后，自由基与氧分子反应生成H_2_O_2_^[26]^。当一个脂基与邻近脂基上另一个双烯丙基上的亚甲基氢发生反应时，可促进脂质过氧化。单个脂肪酸链中的多个双烯丙基也可以发生脂质过氧化。当脂质底物耗尽或者当细胞抗氧化剂减少氧化脂质时，反应就会终止。

细胞膜的组成成分是细胞对铁死亡敏感与否的重要决定因素。在各种类型的细胞脂类中，磷脂（phospholipid, PL）、鞘脂和胆固醇是细胞膜的主要成分。PL的化学多样性由两个脂肪酰基链（sn1和sn2）和头部基团组成。sn1的位置被饱和脂肪酸（saturated fatty acid, SFA）或单不饱和脂肪酸（monounsaturated fatty acid, MUFA）占据，而sn2位点可以是SFA、MUFA或PUFA。膜脂的组成在生物、细胞类型、细胞器甚至膜的子结构域之间高度不同，并且受动态调节。一般来说，富含SFA的PL使细胞膜更刚性，而PL具有较高的不饱和程度，使膜具有一定柔韧性。PUFAs是PL过氧化的底物，虽然增加细胞膜中PUFA-PL的含量可以通过提高细胞的流动性来实现许多细胞功能，但它也增加了内在的对铁死亡的敏感性^[27]^。在动物细胞中，花生四烯酸（arachidonic acid, AA）是最丰富的PUFA之一，AA是一种n-6脂肪酸，含有20个碳链和4个不饱和键（20:4）。由于AA与肾上腺素酸（adrenic acid, AdA）（22:4）都有两个双烯丙基，含AA或AdA的磷脂在sn2位置特别容易发生过氧化^[28]^。在哺乳动物中，这些PLs比其他PUFA-PLs优先氧化，如含有一个双烯丙基的亚油酸（linoleic acid, LA）（18:2）。因此如果细胞膜中有高的PUFA-PL含量，比如神经元，更容易受到过氧化的攻击。

PL过氧化反应可以在细胞内以酶和非酶两种方式启动。脂质的非酶过氧化是由氧化还原活性金属特别是铁催化的。酶促反应包括某些脂氧合酶（lipoxygenase, LOX），是非血红素铁依赖性双加氧酶，可以直接氧化生物膜的PUFAs^[29]^。

### 2.4 细胞对抗铁死亡的还原系统

铁死亡是由铁依赖的磷脂过氧化所驱动的细胞死亡方式，氧和铁是新陈代谢的基本驱动因素，不可避免地产生副产物ROS，由于所有哺乳动物细胞都含有一定水平的PUFA-PLs和生物活性铁，因此需要建立特定的监测或保护机制来保护它们免受有害的铁死亡。

典型的监测机制是由GPX4介导的，近年还发现了一些自由基捕获抗氧化剂（radical-trapping antioxidants, RTA），可以不依赖GPX4减轻铁死亡。例如，脂肪酸合成酶相关蛋白1（fibroblast specific protein 1, FSP1）、鸟氨酸脱羧酶1（GTP cyclohydrolase 1, GCH1）和二氢尿嘧啶脱氢酶（dihydropyrimidine dehydrogenase, DHODH）产生的具有自由基捕获活性的代谢物。

GPX4是一种硒蛋白，研究^[30,31]^也指出了微量元素硒以及维生素E和半胱氨酸可以抑制脂质过氧化。GPX4是已知的哺乳动物中唯一一种催化磷脂氢过氧化物还原为磷脂醇的酶，是催化哺乳动物细胞中PLOOHs减少的主要酶^[32,33]^。GPX4发挥作用需要硒代半胱氨酸残基和主要由谷胱甘肽（glutathione, GSH）提供的两个电子，也由其他低分子硫醇或蛋白质硫醇提供^[34]^。GSH是哺乳动物细胞中最丰富的还原剂，在对抗铁死亡时起着关键作用。GSH以谷氨酸、半胱氨酸、甘氨酸为原料合成，而半胱氨酸则由胱氨酸合成。胱氨酸由谷氨酸出胞通过通道蛋白复合体System Xc按照1:1的比例置换进入细胞。半胱氨酸的含量限制了GSH的合成，胞外谷氨酸浓度过高会影响通道蛋白复合体System Xc的功能，降低胞内半胱氨酸水平从而引起铁死亡。

FSP1原名为凋亡诱导因子线粒体相关2（apoptosis inducing factor mitochondria associated 2, AIFM2），最初被描述为一种促凋亡基因。尽管长链脂肪酸酰基转移酶4（acyl-CoA synthetase long-chain family member 4, ACSL4）被鉴定为促铁死亡基因，其表达决定了铁死亡的敏感性^[35,36]^。但FSP1抗铁死亡功能不依赖于细胞GSH水平、GPX4活性、ACSL4表达和可氧化脂肪酸含量，独立于典型的铁死亡机制^[37]^。FSP1发挥抗铁死亡的作用依赖其N-肉豆蔻酰化。从机理上讲由于凋亡诱导因子（apoptosis inducing factor, AIF）家族成员被证明具有NADH:泛醌氧化还原酶活性^[38]^，还原形式的辅酶Q10（CoQ10H2）在磷脂和脂蛋白中是一种良好的RTA，FSP1在细胞膜上将泛醌（CoQ10）还原为二氢泛醌（CoQ10H2），CoQ10H2作为一种抗氧化剂阻止脂质过氧化，抑制铁死亡发生。FSP1抑制剂与GPX4抑制剂存在协同作用，共同促进了铁死亡的发生，FSP1/CoQ10/NAD(P)H作为一个独立的平行系统存在，它与GPX4和GSH共同抑制磷脂过氧化和铁死亡。

## 3 线粒体在铁死亡中的作用

### 3.1 铁死亡细胞的线粒体形态特征

铁死亡发生时线粒体的形态发生显著变化。电镜下观察到，细胞启动铁死亡后，线粒体整体表现为萎缩，线粒体膜密度增加、嵴减少、退化或消失、外膜破裂。这些都是铁死亡重要且独特的形态学特征。线粒体内膜表面积大，更靠近产生超氧化物的呼吸链，磷脂中尤其是心磷脂不饱和脂肪酸（unsaturated fatty acids, UFAs）含量丰富，更容易受到氧化损伤，而外膜相对不容易受到氧化损伤^[39]^。

### 3.2 线粒体是铁的储存中心与代谢中心

哺乳动物细胞内的大部分铁是作为血红蛋白、铁硫簇的辅助因子以及作为酶的单铁和双铁中心而存在。细胞内的铁大多数储存在线粒体内，线粒体内铁的过量积累促进了ROS的生成，加速了脂质过氧化反应，这与铁死亡的发生发展关系密切。线粒体转运蛋白对铁的调控分工明确，包括摄取铁的蛋白——线粒体铁转运蛋白（mitoferrin, Mfrn）、电压依赖性阴离子通道蛋白（voltage-dependent anion channel, VDAC）、ATP结合盒亚家族B成员1（ATP-binding cassette subfamily B member 1, ABCB1）以及输出铁的蛋白——溶质载体家族25成员37（solute carrier family 25 member 37, SLC25A37）、铁储存蛋白——线粒体铁蛋白（ferritin mitochondrial, FTMT）。除此之外，线粒体内铁硫簇的代谢也与线粒体内铁的调节息息相关，如参与铁硫簇转运的蛋白——ATP结合盒亚家族B成员7（ATP-binding cassette subfamily B member 7, ABCB7）、CIS结构域含量蛋白1（CIS-domain containing protein 1, CISD1）。

Mfrn定位在线粒体膜上，主要负责将细胞外的铁转运到线粒体内，它在铁代谢和血红素合成中发挥关键作用。Mfrn1/2在神经系统疾病中功能受损，如阿尔茨海默病、亨廷顿病和帕金森病，这些疾病都与铁死亡有关^[40]^。

VDAC位于线粒体外膜，主要负责小分子（如ATP、ADP和离子）的运输，它也参与铁的输入。Erastin能够抑制电压依赖性阴离子通道2/3（voltage-dependent anion channel 2/3, VDAC2/3），加速氧化，导致内源性ROS积累^[41]^。

FTMT通过吸收游离铁、三价铁离子并形成铁蛋白复合物来有效储存铁。这一过程不仅有助于调节细胞内铁的稳态，还能防止因铁过量引起的氧化损伤。当细胞需要铁时，FTMT可以通过特定的机制释放储存的铁，以供细胞代谢和生理需求使用。这种结构不仅有效地储存铁，还能防止游离铁引起的氧化应激。研究^[42]^表明FTMT的过表达显著抑制Erastin诱导的铁死亡。

### 3.3 线粒体的能量代谢对铁死亡的影响

线粒体是细胞内氧化呼吸的主要场所，也是ROS的主要来源^[43,44]^。线粒体氧化磷酸化与三羧酸循环中产生的ROS可以作为二级信使来调节各种细胞活性。但肿瘤细胞的高代谢也增加了细胞ROS应激，并使其对铁死亡易感。

肿瘤细胞可通过激活适应性代谢反应来抑制铁死亡，从而实现自我保护。例如，肿瘤细胞会上调糖酵解和磷酸戊糖途径，以提高还原型烟酰胺腺嘌呤二核苷酸磷酸（nicotinamide adenine dinucleotide phosphate, NADPH）和GSH的水平，增强抗氧化能力，减少ROS的积累。这种代谢适应性使肿瘤细胞能够抵抗铁死亡，促进其生存和增殖^[45]^。有氧糖酵解既满足了对能量和生物合成前体的需求，同时抑制线粒体氧化磷酸化活性，减轻了ROS应激。

线粒体氢质子泵（复合物I、III和IV）对产生线粒体膜电位（mitochondrial membrane potential, MMP）至关重要，这一过程对于氧化磷酸化后的能量储存起着关键作用。在细胞凋亡过程中，线粒体膜电位通常会减少。然而，在Erastin或胱氨酸饥饿诱导的铁死亡中，细胞却表现出MMP超极化^[46]^。使用线粒体解偶联剂羰基氰化物间氯苯腙（carbonyl cyanide 3-chlorophenylhydrazone, CCCP）可以破坏MMP，完全阻断脂质ROS的积累和铁死亡，而不会影响长期的细胞活力。铁死亡期间的MMP超极化反映了线粒体电子传递链活性的增强。

不同条件下发生的铁死亡对线粒体代谢需求存在差异。半胱氨酸剥夺诱导的铁死亡或溶质载体家族7成员11（solute carrier family 7 member 11, Slc7a11）抑制剂Erastin诱导的铁死亡依赖线粒体的存在^[46]^。同样阻断线粒体三羧酸循环、电子转移链、谷氨酰胺分解均可减轻MMP超极化、脂质过氧化物积累和铁死亡。但当GPX4被药理学抑制或缺失时，无论线粒体是否缺失以及三羧酸循环、氧化磷酸化如何，细胞都会发生铁死亡^[47]^。这提示GPX4发挥作用抵抗铁死亡位于线粒体下游。支持这一观点的是，线粒体损伤和线粒体ROS产生是在抑制Slc7a11或胱氨酸饥饿后发生的，但对于GPX4抑制诱导的铁死亡不是必需的^[48]^。

### 3.4 线粒体独立的抵抗铁死亡的机制

二氢乳清酸脱氢酶（dihydroorotate dehydrogenase, DHODH）是一种束缚在线粒体内膜外表面的线粒体酶，催化嘧啶生物合成中的第四个酶促步骤，其中DHODH将二氢乳清酸转化为乳清酸盐（嘧啶合成的前体），将CoQ还原为CoQH2，CoQH2可作为自由基捕获抗氧化剂来抑制脂质。重要的是，CoQ主要在线粒体中合成，因此，DHODH将CoQ还原为CoQH2并在线粒体中发挥抗铁死亡的重要作用。

## 4 铁死亡在肿瘤中的作用

铁死亡与肿瘤的关系越来越受关注。首先，一些肿瘤抑制因子，如p53^[49]^和BRCA1相关蛋白（BRCA1-associated protein-1, BAP1）^[50]^抑制肿瘤的机制有铁死亡的参与。其次，越来越多的发现证实抗癌药物存在以铁死亡的方式促进肿瘤细胞死亡。Zhao等^[51]^发现丙泊酚和紫杉醇除促进细胞凋亡外，还可诱导宫颈癌细胞的铁死亡。另外，铁死亡诱导药物与临床药物联用可以增加药物敏感性。Wu等^[52]^研究发现β-榄香烯与埃罗替尼联合，增强了埃罗替尼对原发性耐药非小细胞肺癌细胞的毒性，并降低了铁死亡负调节蛋白[谷氨酰胺酶、铁蛋白重链1（ferritin heavy chain 1, FTH1）、Nrf2、Slc7a11和GPX4]的含量，增加了ROS和脂质过氧化水平，并通过铁死亡实现了抗肿瘤作用。萜类（如双氢青蒿素）、皂苷类（如母皂苷AIII）、生物碱类（如龙葵碱）、黄酮类（如姜黄素）等多种中药成分也被证实参与ROS生成诱导铁死亡，这些中药成分也具有抗肿瘤功效^[53]^。

## 5 研究铁死亡的意义

铁死亡作为一种新发现的细胞死亡形式，在肿瘤与神经系统疾病中有重要意义。在帕金森病、阿尔兹海默病、肌萎缩侧索硬化等神经系统疾病中均发现铁的蓄积与过氧化损伤，通过抑制铁死亡可以保护神经元，改善预后。在肿瘤中，则可以通过促进铁死亡的发生来杀伤肿瘤细胞，或应对肿瘤细胞对化疗、靶向药物耐药的问题。由于铁死亡的发生与铁代谢、脂质过氧化息息相关，而肿瘤细胞的代谢水平明显高于正常细胞，癌细胞相较于正常细胞有更高的ROS水平^[54]^，这使癌细胞对铁死亡更加敏感。越来越多的实验表明诱导肿瘤细胞铁死亡可以改善临床耐药的问题。Feng等^[55]^发现异东方素和顺铂的联合通过控制SIRT6/Nrf2/GPX4信号通路显著降低了耐药细胞的存活能力，调节了铁死亡并逆转了肺癌细胞的耐药性。Lai等^[56]^观察到，双氢青蒿素和吉非替尼的联合治疗对肺腺癌的抑制作用优于单独使用吉非替尼。

## 6 未来与展望

铁死亡作为一种独特的细胞死亡形式有着巨大的研究前景，本文回顾了近年铁死亡的研究进展。随着对铁死亡研究的不断深入，铁死亡的调控机制不断被探明，同时也发现了多种小分子化合物可以靶向诱导铁死亡的发生。但现在对铁死亡的研究多数还停留在细胞层面机制的探索，如何开发出更多的铁死亡靶向药物以及将铁死亡的药物进行临床转化是现在面临的关键问题。由于铁死亡的发生是通过脂质过氧化积累和细胞膜损伤来实现的，因此脂质过氧化物是否会损伤正常细胞以及铁死亡是否会产生其他潜在的副作用仍有待确定。除了铁死亡之外近年还发现了铜死亡等其他金属离子导致的细胞死亡，他们是否也可以引起铁死亡的发生也是一个值得探索的方向。
